# Acute Promyelocytic Leukemia during Pregnancy: A Systematic Review of the Literature

**DOI:** 10.3390/cancers12040968

**Published:** 2020-04-14

**Authors:** Andrea Santolaria, Alfredo Perales, Pau Montesinos, Miguel A. Sanz

**Affiliations:** 1 Department of Obstetrics and Gynecology, Hospital Universitari i Politècnic La Fe, 46026 Valencia, Spain; andrea.santolariaa@gmail.com (A.S.); perales_alf@gva.es (A.P.); 2 Department of Obstetrics and Gynecology, University of Valencia, 46010 Valencia, Spain; 3 Department of Hematology, Hospital Universitari i Politècnic La Fe, 46026 Valencia, Spain; montesinos_pau@gva.es; 4 Centro de Investigación Biomédica en Red de Cáncer, Instituto Carlos III, 28029 Madrid, Spain; 5 Department of Medicine, University of Valencia, 46010 Valencia, Spain

**Keywords:** acute promyelocytic leukemia, pregnancy, all-trans retinoic acid, chemotherapy, arsenic trioxide

## Abstract

The management of pregnant women with acute promyelocytic leukemia (APL) is a challenging situation where limited evidence-based information is available. We performed a systematic literature review to analyze the outcomes reported for both mother and fetus when APL is diagnosed during pregnancy. PubMed, Scopus and Web of Science databases were systematically searched to identify studies reporting cases of APL during pregnancy. Sixty-six articles met the eligibility criteria (53 single case reports). Ninety-two patients were eligible for induction therapy, with most them being treated with all-trans retinoic acid alone (32%) or combined with chemotherapy (43%), while the remaining patients received chemotherapy alone. Three patients were treated with arsenic-based regimens after delivery. Overall complete remission rate was 89%, with no statistically significant differences according to the type of induction and gestational age. During the first trimester, women were more likely to experience spontaneous and induced abortion compared to those during the second trimester (88% vs. 30%) (*p* < 0.0001), while only one patient diagnosed during the third trimester terminated in stillbirth. Twelve of 16 infants with neonatal complications had respiratory distress syndrome. Except two early deaths (Potter’s syndrome and pulmonary hemorrhage), all neonates evolved favorably. This study confirms that gestational age does not affect the results in the mother, but is closely related to fetal viability. Our results may be useful for the process of decision making that requires the involvement of the patient, hematologist, obstetrician and neonatologist.

## 1. Introduction

Acute promyelocytic leukemia (APL) is a subtype of acute myeloid leukemia (AML) with unique molecular pathogenesis, clinical manifestations and treatment. Cytogenetically, it is characterized by a balanced translocation t(15;17) (q24;q21) that generates the PML-RARA fusion gene. This hybrid gene confers a particular sensitivity to treatment with anthracycline-based chemotherapy and differentiating agents, such as all-trans-retinoic acid (ATRA) and arsenic trioxide (ATO), converting this once fatal leukemia into a highly curable disease (cure rates of approximately 90%) [1]. However, the diagnosis and management of APL during pregnancy is a particularly challenging situation. Decision-making in this uncommon clinical scenario is extremely complex according to gestational age, choice of the most suitable therapeutic approach and attitude of the patient towards the increased maternal and fetal risk, sometimes including ethical/moral considerations.

The information available on maternal and fetal outcomes in pregnant women with APL is limited to publications that mostly include a single patient and a few with 2 to 4 patients [2,3], with only one exceeding these figures (14 patients) [2].

The aim of this study is to complete a comprehensive systematic literature review to analyze the clinical outcomes reported for both the mother and the fetus in different scenarios, with particular focus on the use of modern therapies according to gestational age.

## 2. Methods

### 2.1. Search Strategy and Selection of Studies

**Figure 1:**
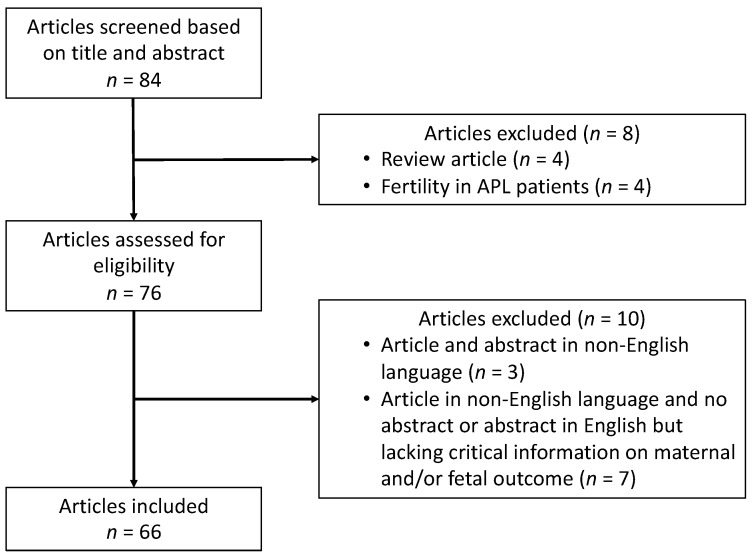
Flowchart of study selection process.

A systematic review of the literature was performed according to the PRISMA guidelines [3] using preset search criteria across PubMed, Scopus and Web of Science databases from inceptions to January 2020. The search was restricted to articles with at least an informative abstract in English using the search terms “acute promyelocytic leukemia” or “acute myeloid leukemia” or “acute leukemia” or “hematologic malignancy” and “pregnancy” or “pregnant”. The search was conducted by two independent reviewers (A.S. and M.A.S.). Once eligible articles were identified, they were further screened and duplicate articles were excluded. A total of 66 articles finally met the eligibility criteria, involving 96 pregnant women who were diagnosed with APL. Figure 1 shows the flowchart of study selection process.

### 2.2. Data Extraction

The following data were extracted from the selected studies: age of the patient at diagnosis, presenting signs and symptoms, blood counts, presence of coagulopathy, genetic diagnosis, type of therapy and response, risk of relapse, gestational age at diagnosis and delivery, type of delivery/abortion, newborn status at birth, including Apgar scores and weight, any obstetric or complications, duration of follow-up and overall clinical status of the mother and newborn at the time of last follow-up.

### 2.3. Statistical Analysis

Statistical analysis was performed using the R software package for Mac (version R 3.6.2). Univariable analysis was performed using the Student *t*-text or Wilcoxon rank sum text for continuous variables, while chi-square or Fisher’s exact tests was used for categorical variables. All *p*-values were two-sided and the level of significance was chosen at 0.05.

## 3. Results

A total of 96 pregnant women who were diagnosed with APL were included in the study. Except for one series of 14 patients, most of patients were reported as single case reports (*n* = 53) and a few articles with 2 to 4 patients (Table 1).

The median age of pregnant women at diagnosis of APL was 30 years (range, 16–41). The median gestational age at diagnosis of APL was 25 weeks (range, 3 to 42). APL was diagnosed during the first, second and third trimester in 16, 46 and 29 women, respectively and immediately after delivery in 4 more patients. Gestational age was unknown in one patient.

The distribution of presenting features, such as white blood cell (WBC) and platelet counts, hemoglobin level, relapse-risk score, coagulopathy and genetic diagnosis is shown in Table 2. At the time of the APL diagnosis, the median WBC and platelet counts was 1.8 × 10^9^/L (range, 0.4–295) and 22 × 10^9^/L (range, 1.5–131), respectively, with thrombocytopenia lower than 40 × 10^9^/L in 48 patients (84%) and hyperleukocytosis higher than 10 × 10^9^/L in 8 patients (14%). Anemia with hemoglobin levels lower than 10 g/dL was present in 36 patients (90%). Patients were classified as low-, intermediate- and high-risk patients in 9 (16%), 40 (71%) and 7 (12%) of 56 women, respectively, in whom this data were available. A vast majority of patients (43 out of 53; 81%) had signs of coagulopathy at presentation. A genetic diagnosis was documented in 68 out of 89 patients (76%) in whom this data were available. No patient had central nervous system involvement.

### 3.1. Maternal Outcome

Except for 4 patients who were admitted in an extremely poor clinical condition and could not receive treatment, the remaining 92 pregnant patients were considered eligible for induction therapy (Table 3). Most patients received induction therapy with ATRA alone (29; 32%) or combined with chemotherapy (40; 44%), while 20 additional patients (22%) received anthracycline-based chemotherapy. Fifteen of the latter had been treated when ATRA was not yet commercially available (1973–1995), while the remaining 5 patients were treated with chemotherapy alone due to physician’s discretion (1995–2002). Three patients were recently treated (2016–2019) with ATO-based regimen after stillbirth at 26 weeks of gestation or delivery of healthy infants at 35 and 39 weeks.

Of the 88 patients who were evaluable for response, 78 (89%) achieved complete remission (CR) and 10 died during induction therapy. The cause of death was described in 7 patients: 3 intracranial hemorrhage, 3 multiorgan failure and 1 infection. Two of the multiorgan failures occurred in a context of severe differentiation syndrome and one ischemic stroke in a Jehovah’s Witness patient that refused blood products. All deaths occurred shortly after diagnosis. Differences in CR rate according to type of induction and gestational age were not statistically significant (Table 3).

### 3.2. Fetal Outcome

Table 4 shows pregnancy outcomes. Overall, 31 pregnancies ended in spontaneous abortion (8; 26%), induced abortion (12; 38%), late stillbirth (8; 26%) or maternal death during pregnancy (2; 3%). In women diagnosed with APL during the first trimester of pregnancy, 14 of 16 (88%) ended in abortion, 9 induced at a median gestational age of 9 weeks (range, 3–11) and 5 spontaneous at a median of 7 weeks of gestation (range, 4–9). The remaining 2 patients continued gestations until delivery of healthy infants by cesarean section at 32 weeks or vaginal delivery at 39 weeks. During the second trimester, 14 of 46 of women (30%) ended their pregnancies in stillbirth (*n* = 7), induced abortion (*n* = 3), miscarriage (*n* = 2) and maternal death during pregnancy with no delivery (*n* = 2). In women diagnosed during the third trimester, there were 2 of 33 pregnancies (6%) who ended in stillbirth or maternal death during pregnancy. The remaining 31 women who were diagnosed during the third trimester (*n* = 27) or immediately after delivery (*n* = 4), delivered by cesarean section (*n* = 17), vaginal delivery (*n* = 13) or unknown route (*n* = 1).

Women diagnosed with APL during the first trimester were more likely to experience spontaneous and induced abortion compared to those diagnosed during the second trimester (88% vs. 30%) (*p* < 0.0001). The proportion of deliveries in pregnant women diagnosed with APL during the third trimester (94%) was significantly higher than those diagnosed in the second (70%) and first trimester (12%) (*p* < 0.0001).

Forty-seven infants were born preterm (28–36 weeks of gestation) and 15 at term (37 weeks of gestation or beyond). Table 5 shows details on birth weight and Apgar score by gestational age at diagnosis of APL in those babies in whom these data were available. The median birth weight was 2200 g (range, 857–3200) and 3124 (range, 2450–4000) for infants born before 36 weeks of gestation and at term, respectively. The median Apgar scores at 1 and 5 min were 6 (range, 2–10) and 9 (range, 4–10), respectively.

Neonatal complications were reported in 16 out of 65 newborns (25%), all of them preterm with a median gestational age of 32 weeks (range, 28–36) and weight of 2000 g (range, 857–2765). Twelve neonates suffered from respiratory distress syndrome. Four of them also had additional complications: blocked atrial premature contractions and arrhythmia, patent ductus arteriosus, pulmonary hypoplasia and cerebral hemorrhage with bilateral hydronephrosis in one patient each. The remaining four infants with neonatal complications had arrhythmia and cardiac arrest that was successfully resuscitated, transient dilated cardiomyopathy, bilateral subependymal hemorrhages and Potter’s syndrome in one patient each. All infants with neonatal complications evolved favorably, except the infant with Potter’s syndrome, who died 30 min after delivery and another weighting 1.05 kg at birth, who developed respiratory distress syndrome and died because of pulmonary hemorrhage after 1 day. Another baby—at last follow up—continued on nasal oxygen and diuretics with significant respiratory effort and poor overall growth. Her developmental progress remained under review at time of publication.

Depending on whether the APL patients had been treated for induction with ATRA alone, chemotherapy alone and ATRA with chemotherapy, the babies with complications at birth were 7/23 (30%), 2/13 (15%) and 6/23 (26%), respectively, but differences were not statistically significant. According to gestational age at diagnosis, the neonatal complications were 1/2 (50%) in the first trimester, 11/32 (34%) in the second and 3/27 (11%), but again these differences were not statistically significant either. Age and other presenting features also showed no association with the occurrence of neonatal complications. As shown in Table 6, we were also unable to demonstrate any relationship between miscarriages and stillbirths with type of induction therapy.

## 4. Discussion

This study shows that, despite the diagnosis of APL in a pregnant woman is a challenging situation, the chances of achieving CR for the mother remain very high, regardless of gestational age at diagnosis. In contrast, fetal viability is strongly related to gestational age, with an abortion rate of 87%, 33% and 7% in those diagnosed of APL during the first, second or third trimester of pregnancy, respectively. Apart from prematurity, respiratory distress syndrome was the most common fetal complication among preterm babies, with apparently no association with the type of antileukemic treatment or other factors.

Given the inability to study prospectively the most appropriate measures for the management of women who are diagnosed with APL during pregnancy, a systematic review of the cases reported in the literature seems the best available and reliable source of evidence-based information to guide decision-making in clinical practice. Although a previous systematic literature review included 43 articles with 71 patients [69], we have been able to significantly increase the number of articles and patients to 66 and 96, respectively. We should emphasize, however, that most studies reported a single patient. Unfortunately, ten articles were not included in the present study because they were written in non-English language or were not sufficiently informative about maternal and fetal outcome: eight single case reports [70,71,72,73,74,75,76,77], one of two case reports [78] and another of nine [79].

Regarding induction failures and causes of death during induction treatment in pregnant women, these were not apparently different from those observed in non-pregnant patients. It is also important to note that while gestational age had no significant impact on the probability of achieving CR in the mother, it was instead crucial in fetal outcomes. In fact, only 2 out of 16 (13%) women diagnosed with APL in the first trimester had a successful pregnancy and delivered normal babies. While one patient, who had been treated with daunorubicin alone for induction followed by cytarabine as maintenance therapy, at 39 weeks of gestation, delivered a normal infant weighing 3050 g [4], the other, treated with ATRA alone, delivered by cesarean section at 32 weeks of gestation a preterm infant weighing 1820 g [5]. It should be noted that the latter patient had been previously informed of the risks, both for herself and for the fetus, rejected the possibility of a therapeutic abortion and expressed the wish to initiate therapy with ATRA. The perinatal course of the baby was characterized by jaundice and respiratory distress syndrome that resolved in 11 days. Although teratogenic effects were not observed, the current recommendations for an early stage of pregnancy is to reject the use of ATRA, due to its teratogenic potential, prioritizing the use of an anthracycline, particularly daunorubicin [80,81].

Knowing that ATRA and chemotherapy seem reasonably safe when given to patients with APL presenting during the second or third trimester of pregnancy [80], it is not surprising that the use of these agents, alone or in combination, was administered to virtually all patients diagnosed with APL in the second and third trimesters, except for a few who were admitted in an extremely poor clinical [2,6,7,64]. In contrast, to avoid a fetal exposure to ATO, this agent was only used in a few patients after delivery [8,57]. When ATO or chemotherapy are needed after delivery, breastfeeding is contraindicated [1,81].

This study confirmed a significantly decreased abortion rate as gestational age increases, with 88%, 30% and 6% of pregnancies ending in abortion during the first, second and third trimester, respectively. In addition, prematurity was relatively frequently observed, with a sizable proportion of premature infants developing respiratory distress syndrome. Except one, all infants with this neonatal complication, by far the most commonly reported, evolved favorably. A few other neonatal complications were really very scarce and transitory, except the fetus with Potter’s syndrome (oligohydramnios and bilateral renal agenesis) developed before diagnosis with APL and, therefore, before starting induction therapy [9]. The lack of teratogenic effects reported in neonates should be emphasized.

## 5. Conclusions

This study represents to date the most comprehensive literature review on the topic and provides evidence that can help in decision making when facing the situation of a pregnant woman who is diagnosed with APL. In fact, the chances of achieving CR and then of cure, remain very high in this setting and probably not very different than in non-pregnant patients. Fetal outcome is, however, strongly related to gestational age, with a statistically significant increase of abortion rate in early pregnancies. Prematurity and low birth weight were relatively frequent, with respiratory distress syndrome being the most common fetal complication among preterm babies. Despite the lack of teratogenic effects reported in neonates, the use of potentially teratogenic agents, such as ATRA, chemotherapy and ATO, should be done judiciously according to gestational age, according to the current recommendations from expert panels [1,80,81]. Keeping in mind these recommendations, the process of decision making requires the involvement of the patient, hematologist, obstetrician and neonatologist.

## Figures and Tables

**Table 1:** Case reports of acute promyelocytic leukemia (APL) during pregnancy included in the study.

No. of Patients Reported by Article	No. of Articles	No. of Patients	References
One	53	53	[4,5,6,7,8,9,10,11,12,13,14,15,16,17,18,19,20,21,22,23,24,25,26,27,28,29,30,31,32,33,34,35,36,37,38,39,40,41,42,43,44,45,46,47,48,49,50,51,52,53,54,55,56]
Two	7	14	[57,58,59,60,61,62,63]
Three	4	11 *	[64,65,66,67]
Four	1	4	[68]
More than four	1	14	[2]

* One of 3 cases reported by Takitani et al. had been previously reported as single case report by Terada et al.

**Table 2:** Characteristics of patients with APL presenting during pregnancy.

Characteristic		Median (Range)	No. (%)
Overall			96 (100)
Age, year		30 (16–41)	
	16–20		8 (8)
	21–30		44 (46)
	31–40		41 (43)
	41		3 (3)
Gestational age at diagnosis (*n* = 95)		25 (1–42)	
	First trimester		16 (17)
	Second trimester		46 (48)
	Third trimester		29 (31)
	After delivery		4 (4)
WBC count, ×10^9^/L (*n* = 57)		1.8 (0.4–295)	
	Less than 5		44 (77)
	5–10		5 (9)
	10–50		4 (7)
	50 or higher		4 (7)
Platelet count, ×10^9^/L (*n* = 57)		22 (1.5–131)	
	Less than 40		48 (84)
	40 or higher		9 (16)
Hemoglobin, g/dL (*n* = 40)		8.3 (3.2–12)	
	Less than 10		36 (90)
	10 or higher		4 (10)
Risk score (*n* = 56)			
	Low		9 (16)
	Intermediate		40 (71)
	High		7 (12)
Coagulopathy (*n* = 53)			
	No		10 (19)
	Yes		43 (81)
Genetic diagnosis (*n* = 90)			
	No		22 (24)
	Yes		68 (76)

Percentages may not sum to 100 because of rounding.

**Table 3:** Induction therapy and results in pregnant women with APL by gestational age at diagnosis.

Induction Therapy.		No. Patients (%)	CR/No. patients (%)			
			First Trimester	Second Trimester	Third Trimester	Total *
Total		92 (100)	16/16 (100)	37/44 (84)	25/28 (89)	78/88 (89)
	Chemotherapy alone	20 (22)	5/5 (100)	8/9 (89)	3/4 ^+^ (75)	16/18 (89)
	ATRA alone	29 (32)	2/2 (100)	10/14 (71)	10/11 (91)	22/27 (81)
	ATRA + Ida/Dauno	31 (34)	6/6 (100)	13/14 (93)	11/11 (100)	30/31 (97)
	ATRA + Chemotherapy	9 (10)	3/3 (100)	5/6 (83)	-	8/9 (89)
	ATO ± ATRA ± Chemotherapy	3 (3)	-	1/1 (100)	1/2 (50)	2/3 (67)

CR: complete remission; ATRA: all-trans retinoic acid; ATO: arsenic trioxide; Ida: idarubicin; Dauno: daunorubicin. * The induction response was not available in 2 patients treated with chemotherapy alone and 2 with ATRA alone. ^+^ One patient was diagnosed after delivery. Percentages may not sum to 100 because of rounding.

**Table 4:** Pregnancy outcomes by gestational age at diagnosis in pregnant women with APL.

Pregnancy Outcome		Overall	First Trimester			Second Trimester			Third Trimester		
			No. of Patients (%)	Gestational Age at Diagnosis, wks	Gestational Age at Delivery/Abortion, wks	No. of Patients (%)	Gestational Age at Diagnosis, wks	Gestational Age at Delivery/Abortion, wks	No. of Patients (%)	Gestational Age at Diagnosis, wks	Gestational Age at Delivery/Abortion, wks
Delivery		65 (68)	2 (12)			32 (70)			31 (94)		
	Cesarean	37 (58)	1 (50)	4	32	19 (59)	24 (13–28)	31 (25–40)	17 (55)	33 (29–38)	33 (32–39)
	Vaginal	26 (39)	1 (50)	8	39	12 (38)	23 (13–28)	32 (26–37)	13 (42)	38 (29–42)	38 (29–42)
	Unknown	2 (3)				1 (3)	25	28	1 (3)	29	32
Abortion		31 (32)	14 (88)			14 (30)			2 (6)		
	Spontaneous *	8 (26)	5 (36)	7 (4–9)	7 (6–12)	2 (14)	14, 19	19, 19			
	Therapeutic	12 (38)	9 (64)	9 (3–11)	9 (5–18)	3 (21)	13 (13–14)	15 (13–17)			
	Late stillbirth	8 (26)				7 (50)	26 (23–28)	26 (25–30)	1 (50)	29	29
	Maternal death during pregnancy	3 (10)				2 (14)	25, 28	25, 28	1 (50)	29	29

* Gestational age is missing in one spontaneous abortion.

**Table 5:** Birth weight and Apgar score of infants by gestational age at diagnosis.

Trimester of Pregnancy	Weight at Birth, g						Apgar Score					
	Preterm			At Term			1 min			5 min		
	*n*	Median	Range	*n*	Median	Range	*n*	Median	Range	*n*	Median	Range
First trimester	1	1820		1	3050		0	-			-	
Second trimester	23	1975	857–2950	0		-	12	6	2–9	12	7	4–10
Third trimester	9	2045	1634–3200	8	3124	2450–4000	11	8	6–10	10	10	7–10
Overall	33	2200	857–3200	9	3124	2450–4000	23	6	2–10	22	9	4–10

**Table 6:** Relationship between induction therapy and miscarriage/stillbirth by gestational age at diagnosis.

Induction therapy	No. Patients Who Achieved CR	Miscarriage/Stillbirth *			
		First Trimester	Second Trimester	Third Trimester	Total
**Total**	78	5/0	0/4	0/2	5/6
Chemotherapy alone	16	1/0	-	0/1	1/1
ATRA alone	22	-	0/1	0/1	0/2
ATRA + Ida/Dauno	30	2/0	0/2	-	2/2
ATRA + Chemotherapy	8	2/0	-	-	2/0
ATO ± ATRA ± Chemotherapy	2	-	0/1 ^+^	-	0/1

CR: complete remission; ATRA: all-trans retinoic acid; ATO: arsenic trioxide; Ida: idarubicin; Dauno: daunorubicin. * Induced abortions were not included. ^+^ Treatment with ATO was started after examination revealed intrauterine fetal demise and placental abruption.
